# High concentrations of soluble endoglin can inhibit BMP9 signaling in non-endothelial cells

**DOI:** 10.1038/s41598-023-33352-3

**Published:** 2023-04-24

**Authors:** Clara Andersson-Rusch, Bin Liu, Ingrid Quist-Løkken, Paul D. Upton, Oddrun Elise Olsen, Hanne Hella, Xudong Yang, Zhen Tong, Nicholas W. Morrell, Toril Holien, Wei Li

**Affiliations:** 1 Department of Clinical and Molecular Medicine, Norwegian University of Science and Technology (NTNU), 7491 Trondheim, Norway; 2 Department of Hematology, St. Olav’s University Hospital, Trondheim, Norway; 3 Department of Medicine, Victor Phillip Dahdaleh Heart and Lung Research Institute, School of Clinical Medicine, University of Cambridge, Papworth Road, Cambridge Biomedical Campus, Cambridge, CB2 0BB UK; 4 Department of Biomedical Laboratory Science, NTNU, Trondheim, Norway; 5 Department of Immunology and Transfusion Medicine, St. Olav’s University Hospital, Trondheim, Norway

**Keywords:** Cell biology, Cell signalling, Extracellular signalling molecules, Growth factor signalling, Morphogen signalling

## Abstract

Endoglin (ENG) is a single-pass transmembrane protein highly expressed on vascular endothelial cells, although low expression levels can be detected in many other cell types. Its extracellular domain can be found in circulation known as soluble endoglin (sENG). Levels of sENG are elevated in many pathological conditions, in particular preeclampsia. We have shown that while loss of cell surface ENG decreases BMP9 signaling in endothelial cells, knocking down ENG in blood cancer cells enhances BMP9 signaling. Despite sENG binding to BMP9 with high affinity and blocking the type II receptor binding site on BMP9, sENG did not inhibit BMP9 signaling in vascular endothelial cells, but the dimeric form of sENG inhibited BMP9 signaling in blood cancer cells. Here we report that in non-endothelial cells such as human multiple myeloma cell lines and the mouse myoblast cell line C2C12, both monomeric and dimeric forms of sENG inhibit BMP9 signaling when present at high concentrations. Such inhibition can be alleviated by the overexpression of *ENG* and *ACVRL1* (encoding ALK1) in the non-endothelial cells. Our findings suggest that the effects of sENG on BMP9 signaling is cell-type specific. This is an important consideration when developing therapies targeting the ENG and ALK1 pathway.

## Introduction

Endoglin (ENG), also known as CD105, is a single-pass transmembrane glycoprotein that is highly expressed by endothelial cells (EC). It plays a critical role in regulating cardiovascular development and vascular remodeling^1^. Loss-of-function mutations in ENG cause type I hereditary haemorrhagic telangiectasia (HHT1). Symptoms of HHT include telangiectases in the nose, gastrointestinal tract and skin, as well as larger, life-threatening arteriovenous malformations (AVMs) in the brain, lungs and liver. ENG mutations were also found in patients with pulmonary arterial hypertension (PAH), a vascular disorder characterized by the remodeling of small pulmonary vessels, leading to increased right ventricular systolic pressure and ultimately right-sided heart failure.

ENG has 561 amino acids (aa) in the extracellular domain (ECD) and 47 aa in the intracellular domain (ICD). A short form of ENG has been detected in human cells containing the same ECD sequence but only 14 aa in the ICD. The ECD can be shed from the cell surface under conditions related to endothelial dysfunction and inflammation. Cleaved ENG ECD, also known as soluble endoglin (sENG), is markedly elevated in the maternal circulation in preeclampsia (PE) and is thought to contribute to the pathogenesis of the disease, although the mechanism is poorly understood.

Other than its role in endothelial cells, ENG expression has also been found on chondrocytes and cells in the hematopoietic niche such as mesenchymal stem cells (MSC) and monocytes^2,3^, implying a role for ENG in immune cell differentiation and function. In cancer, opposing roles for ENG have been described^4^. ENG can modify the microenvironment, promote the formation of new blood vessels and contribute to tumor progression and metastasis, although tumor-suppressing roles have also been suggested^4^. In solid as well as hematological cancers, expression of ENG on the cell surface of tumor cells is associated with disease progression^5^. In patients with the bone marrow cancer multiple myeloma, sENG was elevated in serum samples compared to normal controls and high levels of sENG predicted poor prognosis^6,7^. Bone morphogenetic protein 9 (BMP9) induces apoptosis in multiple myeloma cells and sENG is one of few known circulating factors that antagonizes such an activity of BMP9. Both BMP9 and sENG are present in bone marrow plasma from multiple myeloma patients, and both soluble and cell-surface ENG inhibited BMP9-induced apoptosis^8^.

Although ENG was discovered as a co-receptor for TGF-β signaling, it was found later that its ECD only binds to BMP9 and BMP10 with high affinity. BMPs constitute the largest subgroup of the TGF-β family of ligands and due to their pleiotropic functions, they have also been called body morphogenetic proteins^9,10^. Their roles span from embryonic development and adult tissue homeostasis to major roles in human diseases^11,12^. BMPs initiate cell signaling by forming complexes comprising two copies of a type I receptor and two copies of a type II receptor^13^. The active ligand-receptor complex enables phosphorylation of SMAD1/5/8 transcription factors that regulate the transcription of a variety of genes, depending on cell type and the different cofactors involved.

The ENG binding site on BMP9 overlaps with the type II receptor binding site^14,15^. How ENG participates in BMP9 signaling is not fully understood. We have previously shown that loss of cell surface ENG in endothelial cells led to reduced BMP9 signaling^16^, but siRNA knocking down of ENG in a blood cancer cell line enhanced BMP9-induced SMAD1/5 signaling^8^. Interestingly, BMP9 also has an opposite effect on apoptosis between endothelial cells and blood cancer cells. While BMP9 protects vascular endothelial cells from apoptosis induced by serum starvation or combined treatment of TNFα and cycloheximide^17^, both BMP9 and BMP10 inhibit growth and induce apoptosis in multiple myeloma cells^8,18^.

The type I receptors for BMP9 signaling can be activin receptor-like kinase 1 (ALK1) or ALK2. BMP9 binds ALK1 with particularly high affinity; therefore if a cell expresses both ALK1 and ALK2 the ligand preferentially signals through ALK1 at low concentrations. At high concentrations or in the absence of ALK1, BMP9 also signals through ALK2. Because ALK1 is highly expressed in vascular endothelial cells, BMP9 signaling in endothelial cells is almost exclusively mediated by ALK1^19^. In cancer cells, BMP9 signaling is mostly mediated by ALK2^8,20,21^. We have reported that sENG does not inhibit BMP9 signaling in wild type endothelial cells, but it inhibits BMP9 signaling in *Eng*-/- endothelial cells and in blood cancer cells^16^. To understand what factors influence sENG function in different cells, here we investigate the effect of sENG on BMP9 signaling in different non-endothelial cells including cancer cells, and whether cell surface ENG and ALK1 expression influence the cellular response to sENG and BMP9.

## Results

### Soluble forms of ENG inhibit BMP9-mediated signaling and apoptosis in myeloma cells

**Figure 1:**
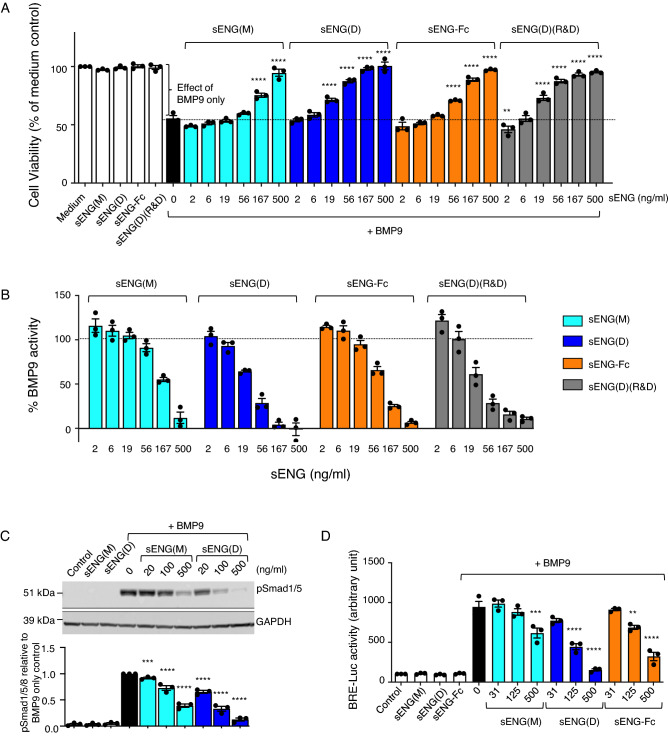
Soluble endoglin (sENG) counteracts BMP9-mediated signaling and apoptosis in myeloma cells. (**A**&**B**) INA-6 cells were treated with BMP9 (0.125 ng/mL) combined with increasing doses of recombinant sENG variants. Cell viability was measured after 48 h using the CellTiter Glo ATP assay. The experiments were performed three times, with duplicated measurement taken in each experiment. (**A**) Readout of Cell viability as percentage to medium only without treatment. (**B**) %BMP9 activity derived from (A), calculated by the difference between BMP9 treatments and the medium treatment and normalizing the values to the effect of BMP9 only treatment as indicated in (A), using the following equation: 100% x (Viability_medium_ − Viability_BMP9+sENG_)/ (Viability_medium_ − Viability_BMP9 only_). **(C)** INA-6 cells were treated with BMP9 (0.5 ng/mL) and increasing doses of sENG(M) or sENG(D) for 1 h. Phosphorylation of SMAD1/5/8 (pSMAD1/5/8) was measured by western blot with GAPDH as a loading control. One representative gel from three independent experiments is shown (upper panel), and the relative pSMAD1/5 levels were calculated by correcting to GAPDH loading control and normalizing to BMP9-treated control (lower panel). Uncropped gels are shown in Supplementary Fig. 1B. (**D**) INA-6 BRE-Luc cells were treated for 18 h with BMP9 (0.4 ng/mL) and increasing doses of sENG(D), sENG(M) or sENG-Fc before the amount of luciferase was measured. In Panels A, C and D, the concentrations of sENG only were 500 ng/mL; the graphs represent Mean ± SEM from N = 3 independent experiments; One-way ANOVA of the BMP9-treated groups, with Dunnett’s multiple comparison against BMP9 alone without sENG (black bar). **P < 0.01, ***P < 0.001, ****P < 0.0001.

Both BMP9 and BMP10 induce growth arrest and/or apoptosis in multiple myeloma cells in a SMAD1/5/8-dependent manner^8,18^. The dimeric form of soluble ENG, sENG(D), inhibited BMP9-induced SMAD1/5 phosphorylation and apoptosis in vitro^8^. However, monomeric ENG, sENG(M), may be the most biologically relevant soluble form in vivo*;* both sENG(D) and sENG(M) were found to facilitate, not inhibit, BMP9-mediatied signaling in endothelial cells^16^. BMP9 signaling in endothelial cells is mediated by ALK1. Multiple myeloma cells do not express ALK1, and they respond well to BMP9 via ALK2^8^. Therefore we set out to test the effect of different forms of sENG on BMP9 signaling in the ALK2-expressing myeloma cell line INA-6. Treating INA-6 cells with 0.125 ng/ml of BMP9 reduced the number of viable cells by approximately 50% (Fig. 1A) and we previously showed that BMPs reduce cell numbers in this cell line primarily via induction of apoptosis as shown by annexin V staining and caspase-3 cleavage^22^. Addition of various forms of sENG with BMP9 resulted in sENG antagonizing BMP9-induced apoptosis in a dose-dependent manner (Fig. 1A). The effects of sENG were clearly shown when displaying this data as %BMP9 activity (Fig. 1B). The monomeric variant, sENG(M), also antagonized BMP9 activity, but the efficiency was lower. We also performed this assay in a second myeloma cell line, IH-1, that expresses both ALK2 and ALK3 and hence responds to both BMP9 and BMP10^18,23^. In this cell line, both BMP9- and BMP10-induced apoptosis was inhibited by sENG in a similar way, with sENG(D) being more efficient than sENG(M) (Supplementary Fig. 1A). To confirm the inhibitory effects of sENG on BMP9 signaling in myeloma cells, we performed signaling assays in INA-6 cells. Both sENG(M) and sENG(D) dose-dependently antagonized BMP9-induced SMAD1/5 phosphorylation (Fig. 1C, Supplementary Fig. 1B). In a BMP-responsive element-luciferase (BRE-luc) reporter gene assay^24^, the same BMP9-inhibitory effects were observed for both sENG(M) and sENG(D) (Fig. 1D). Similar to the ATP cell viability assay, sENG(M) was less effective than sENG(D) in antagonizing BMP9 activity in both signaling assays.

### sENG inhibits BMP9 activity in other non-endothelial cells

**Figure 2:**
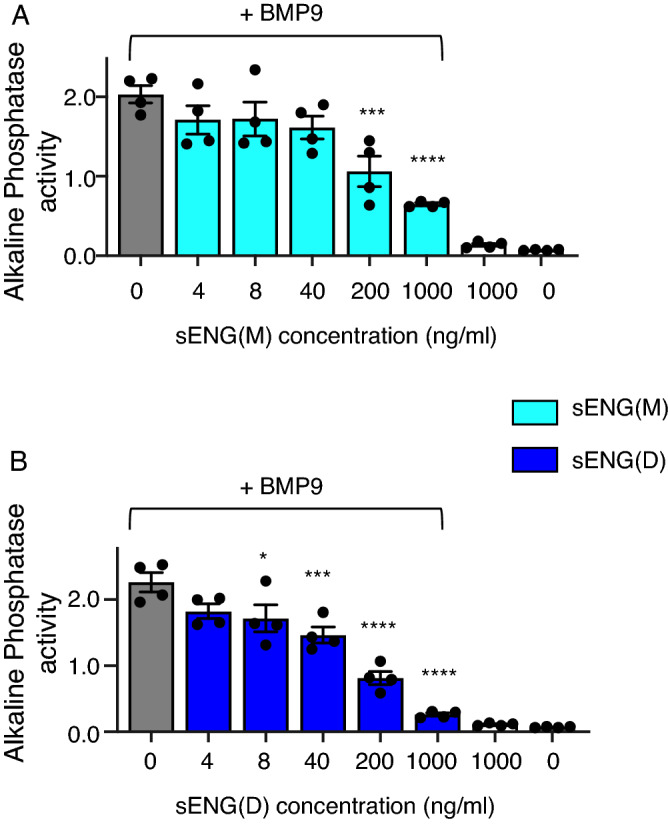
sENG counteracts BMP9-induced alkaline phosphatase (ALP) activity in C2C12 cells. C2C12 cells were seeded in 24-well plated till about 80% confluent before applying BMP9 (10 ng/ml) with or without sENG(M) or sENG(D) for 65 h. Cells were lysed on ice and cell lysates were quantified and ALP activity determined as described in the Methods. The graphs represent Mean ± SEM from N = 3 independent experiments. One-way ANOVA of the BMP9-treated groups, with multiple comparison of effect of sENG to no sENG control. *P < 0.05, ***P < 0.001, ****P < 0.0001.

To ascertain whether the BMP9-inhibitory activity by sENG is specific to myeloma cancer cells or they also apply to other non-endothelial cells that do not express ALK1, we evaluated the effects of sENG on BMP9-induced expression of early osteogenic marker alkaline phosphatase (ALP) in C2C12 myoblast cells. This is a well-documented BMP9 activity in a non-endothelial cell line. As shown in Fig. 2 and consistent with the results in myeloma cells, both forms of sENG inhibited BMP9-induced ALP activity, with sENG(D) being more potent than sENG(M). This suggests that the ability of sENG to inhibit BMP9-signaling may be dependent on the different repertoire of BMP type 1 receptors expressed in these non-endothelial cells.

### sENG inhibits BMP9 binding to the type II receptor ActRIIA, but not the type I receptor ALK1 in a cell-free assay

**Figure 3:**
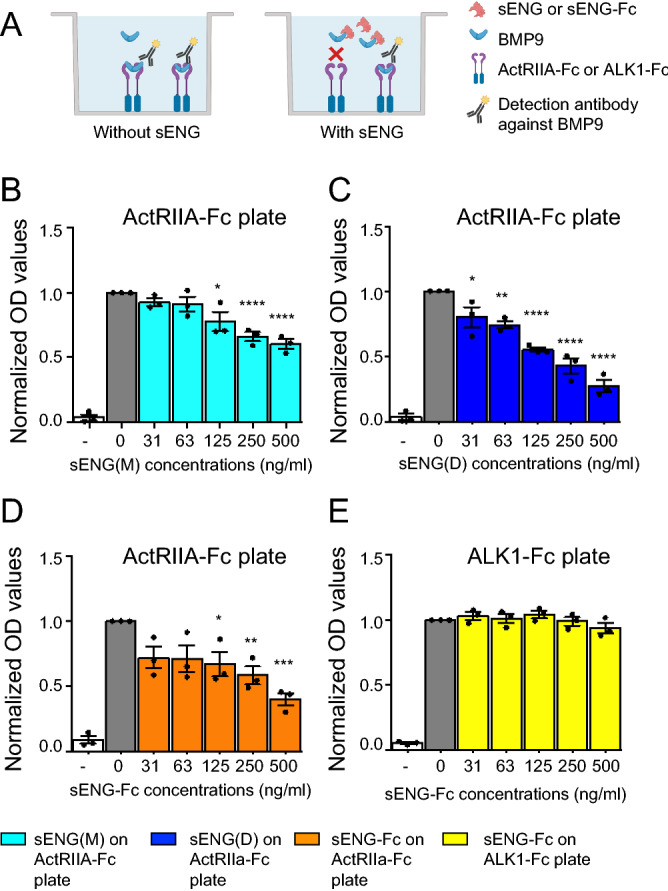
sENG inhibits BMP9 binding to the type II receptor ActRIIA but not the type I receptor ALK1. (**A**) Schematic showing the ELISA set-up where BMP9 is captured using Fc-fusion receptors (ActRIIA-Fc or ALK1-Fc). Binding of BMP9 is detected with a BMP9 specific, enzyme-conjugated antibody. The different added sENG variants compete with BMP9 to give a concentration-dependent reduction in the antibody signal. Created with BioRender.com. (**B-D**) ELISA plates were coated with type II receptor ActRIIA-Fc, followed by incubating with BMP9 (15 ng/mL) alongside increasing concentrations of sENG(M) (**B**), sENG(D) (**C**) or sENG-Fc (**D**). ALK1-Fc coated plate was used as a control (**E**). The graphs represent Mean ± SEM from N = 3 independent experiments. One-way ANOVA with multiple comparison of effect of sENG to no sENG control. *P < 0.05, **P < 0.01, ***P < 0.001, ****P < 0.0001.

BMP9 has a high affinity for ALK1 but also binds other receptors including the three type II receptors (BMPRII, ActRIIA and ActRIIB) with variable affinities^25,26^. In myeloma cells that do not express ALK1, BMP9 is dependent on ALK2 for signaling as shown using CRISPR/Cas9 to knock out ALK2 in INA-6 cells (Supplementary Fig. 2). Although BMP9 may use ALK2 for signaling, the affinity of ALK2 for BMP9 is very weak in the absence of cofactors. To further analyze how sENG(D) and sENG(M) impact on ligand-receptor interaction, we performed ELISA binding assays with recombinant Fc-receptors coated on the plates as substrate and BMP9 as the analyte as shown earlier^27^ (Fig. 3A). BMP9 binding to type II receptor ActRIIA was dose-dependently inhibited by all tested forms of sENG, with sENG-Fc and sENG(D) being more efficient than sENG(M) (Fig. 3B–D). The control experiment shows sENG did not inhibit binding of BMP9 to ALK1-Fc (Fig. 3E), consistent with ALK1 and sENG binding to different sites on BMP9. These results support that various forms of sENG can inhibit the interaction between BMP9 and ActRIIA.

### Effect of ENG transfection on BMP9 signaling in the presence and absence of sENG

**Figure 4:**
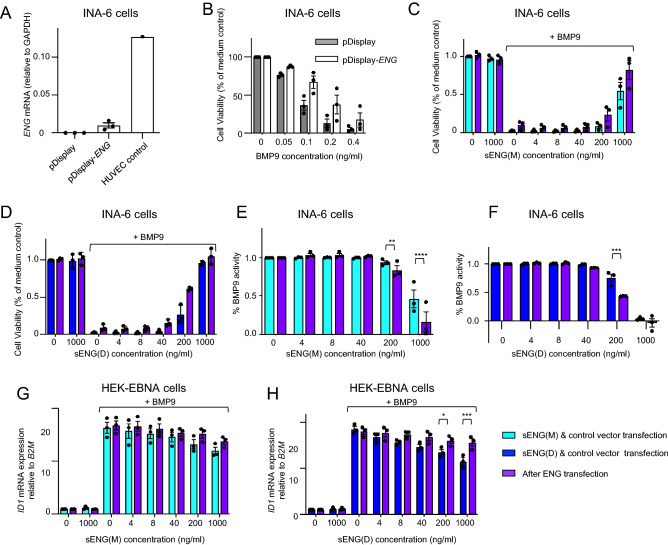
Effect of ENG transfection on BMP9 signaling in the presence or absence of sENG. (**A**–**F**) ENG in stably transfected myeloma cells inhibits BMP9-induced cell death and signaling. (**A**) ENG mRNA levels were measured by RT-qPCR in INA-6 cells stably transfected with pDisplay vector control or pDisplay-ENG; (**B**) Cells stably transfected with pDisplay and pDisplay-ENG were treated with increasing doses of BMP9. Cell viability was measured after 72 h using CellTiter Glo ATP assay and plotted as % of medium-treated control. **(C**–**F)** INA-6 cells stably transfected with pDisplay or pDisplay-ENG were treated with BMP9 (0.5 ng/mL) with increasing doses of sENG(M) **(C**&**E**) or sENG(D) (**D**&**F**). The cell viability relative to medium control **(C**&**D**) and the calculated % BMP9 activity compared to cells treated with BMP9 only (**E**&**F**) are shown. % BMP9 activity is calculated as described in Fig. 1B. (**G**&**H**) Effect of sENG on BMP9-induced *ID1* gene induction in HEK EBNA cells with or without ENG transfection. HEK EBNA cells transfected with vector control or pDisplay-ENG were treated with 3 ng/ml BMP9 for 1.5 h in the presence or absence of sENG(M) or sENG(D) at indicated concentrations. Cells were then harvested for RNA extraction. Relative changes in *ID1* gene expression were measured by RT-qPCR using the ∆∆CT methods. Three independent transfection experiments were performed, and data shown as Mean ± SEM. For **E** to **I**, Two-way ANOVA comparing sENG effect on BMP9 signaling in receptor-transfected and vector control-transfected cells, *P < 0.05, **P < 0.01, ***P < 0.001, ****P < 0.0001.

We have previously shown that although sENG does not inhibit BMP9 signaling in wild type endothelial cells, it does suppress BMP9 signaling in mouse *Eng* -/- endothelial cells^16^. ENG is rarely expressed in myeloma cells. To evaluate whether the presence of cell surface ENG changes the effects of sENG on BMP9 signaling, we generated an INA-6 cell line stably transfected with full-length ENG, which is the membrane-bound form. Successful transfection of ENG was confirmed by RT-qPCR (Fig. 4A), albeit the expression is still very low compared to the HUVEC control. Such ENG-expressing cells were less sensitive to BMP9-induced apoptosis than cells transfected with the control plasmid (Fig. 4B), suggesting that cell surface ENG expression may antagonize BMP9 in non-endothelial cells (that do not simultaneously express ALK1). Interestingly, both sENG(M) and sENG(D) can still inhibit BMP9 activity in the ENG-transfected INA-6 cells (Fig. 4C–F).

Because the expression of ENG in the transfected INA-6 cells is very low and we cannot detect ENG proteins by western blot, we sought to find an alternative non-endothelial cell line that can express membrane-bound full-length ENG to levels comparable to endothelial cells. Thus, we chose HEK EBNA cells which is a cell line commonly used for recombinant protein production. Immunoblot showed that ENG expression in this cell line is dependent on plasmid concentrations; and can achieve levels even higher than the endogenous ENG in pulmonary arterial endothelial cells (hPAECs, Supplementary 3A). We chose 1 μg of plasmid in the transfection to express full-length ENG at a level similar to that in hPAECs, and evaluated the effects of sENG on BMP9 signaling. BMP9 potently induced *ID1* gene expression in HEK EBNA cells (Fig. 4G,H). Both sENG(M) and sENG(D) can inhibit BMP9 activity in these cells but only at very high sENG concentrations. ENG overexpression in HEK EBNA cells reduced the sENG inhibitory effects on BMP9 signaling, with the biggest effect seen at the highest concentration of sENG(D).

### sENG(M) does not inhibit BMP9 activity in ALK1-expressing non-endothelial cells

**Figure 5:**
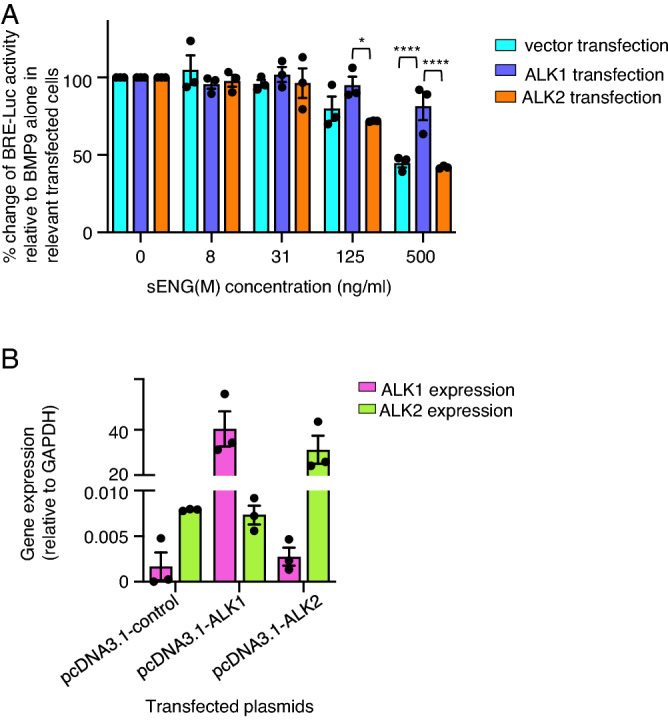
ALK1 but not ALK2 transfection reduced the inhibitory effect of sENG on BMP9 signaling in C2C12 BRE luciferase assay. (**A**) C2C12 BRE-luc cells were transfected with pcDNA3.1-control (cyan bars), pcDNA3.1-ALK1 (blue-purple bars) or pcDNA3.1-ALK2 (orange bars) and treated with BMP9 (1 ng/ml) combined with increasing doses of sENG(M). Cells were treated for 18 h and luciferase activity was measured and normalized to the BMP9 only treatment controls in the relevant transfected cells. Three independent transfection experiments were performed, and data shown as Mean ± SEM. Two-way ANOVA comparing sENG effect on BMP9 signaling in receptor-transfected and vector control-transfected cells, *P < 0.05, ****P < 0.0001. (**B**) Relative gene expression of *ALK1* and *ALK2* measured by RT-qPCR in transfected cells. Gene expression relative to GAPDH from three independent experiments are plotted.

We hypothesized that ALK1 expressed by endothelial cells might be essential for their response to sENG. To test this, we transfected ALK1- or ALK2-containing plasmids into BRE-luc C2C12 myoblast cells. The cells were then treated with BMP9 (1 ng/ml) in the absence or presence of increasing concentrations of sENG(M) and the luciferase activity was measured. The percentage change of BMP9 activity relative to BMP9 alone treatment in each transfection condition were calculated and plotted. As expected, in control vector transfected cells and cells expressing ALK2, sENG inhibited BMP9-induced luciferase activity at concentrations higher than 125 ng/ml (Fig. 5A). Interestingly, in cells expressing ALK1, sENG(M) did not inhibit BMP9-induced luciferase activity even at 500 ng/ml. The successful transfection of ALK1 and ALK2 was confirmed by monitoring ALK1 and ALK2 mRNA using RT- qPCR (Fig. 5B).

### Introducing ENG and ALK1 into C2C12 myoblast cells reduces sENG-mediated inhibition of BMP9-induced ALP activity

**Figure 6:**
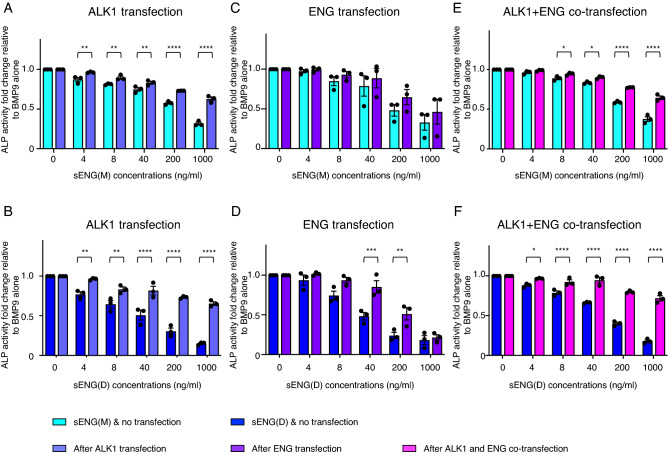
Transfecting full-length ALK1 and ENG reduces the inhibitory effect of sENG on the BMP9-induced ALP activity in C2C12 cells. C2C12 cells were transfected with pcDNA3.1-ALK1 (blue-purple bars), pDisplay-ENG (purple bars), or both (magenta bars), and treated with BMP9 (10 ng/ml) in the presence of different concentrations of sENG(M) or sENG(D). ALP assays were performed as described in the methods. Three independent transfection and treatment experiments were performed. Data were normalized to the BMP9 only treatment in each experiment and Mean ± SEM are shown. Two-way ANOVA comparing sENG effect on BMP9 signaling in receptor-transfected and vector control-transfected cells. *P < 0.05, **P < 0.01, ***P < 0.001, ****P < 0.0001.

We next investigated the inhibitory effects of sENG on BMP9-induced ALP activity in C2C12 cells, without or with transfection of full-length ALK1, full-length ENG or both (Fig. 6). Expression of ENG and ALK1 after transfection was confirmed by immunoblotting (Supplementary Fig. 4). Compared with HEK EBNA cells, it is difficult to achieve high ENG expressions even using 5 times higher DNA concentrations, whereas transfection of ALK1 can achieve similar expression levels as the levels in endothelial cells. Nevertheless, we compared the effect of sENG in the ENG- and ALK1-transfected C2C12 cells with control vector-transfected cells. In ALK1-transfected C2C12 cells, both the monomeric and the dimeric sENG were less effective in inhibiting of BMP9-induced ALP activity (Fig. 6A,B). Overexpression of ENG in C2C12 cells gave rise to a similar result as ALK1-transfection but with less pronounced effect than ALK1 overexpression (Fig. 6C,D). Co-transfecting both ALK1 and ENG showed a similar result as ALK1-transfection alone (Fig. 6E,F). The reason we did not observe additive effects from ALK1 and ENG co-transfection could be due to either that overexpression of ALK1 has the dominant effect, or that transfected ENG level is too low to show any additional effect from ALK1-transfection alone.

### Effect of sENG on BMP9 signaling is context-dependent

**Figure 7:**
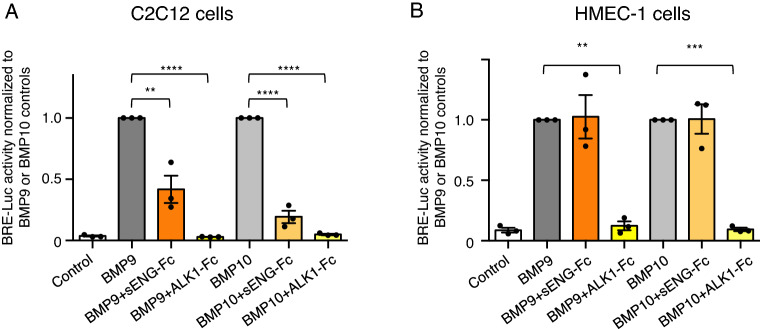
The effect of sENG is context dependent. Parallel luciferase assays were performed using BMP-response element (BRE) transfected non-endothelial cells C2C12 or endothelial cell line HMEC-1, with BMP9 or BMP10 treatment, in the presence or absence of sENG-Fc. ALK1-Fc was used as a positive control. BMP9 or BMP10 (both at 0.2 ng/ml for HMEC-1 cells and 10 ng/ml for C2C12 cells) were incubated with sENG-Fc (2.5 μg/ml) or ALK1-Fc (1 μg/ml) at 37 °C overnight before applying to the cells. Data are Mean ± SEM from three independent experiments. For each experiment, triplicate treatments were performed and data averaged, normalized to BMP9 or BMP10-treated controls, respectively. One way ANOVA analyses were performed in each BMP9 or BMP10 treated groups. **P < 0.01, ***P < 0.001, ****P < 0.0001.

Finally, we evaluated the effect of sENG on BMP9 signaling using BRE-Luc assays in two cell lines both stably transfected with BRE-Luc reporter gene. One is the HMEC-1 cells which is an endothelial cell line with high levels of ALK1 and ENG expressed on the cell surface, the other is C2C12 cells without detectable ALK1 and ENG expression as discussed above. This allowed us to compare the effect of sENG on BMP9 signaling using the same assay but in two cell lines naturally different in ALK1 and ENG expression. sENG-Fc which showed the most potent BMP9 inhibitory activity (Fig. 1) was used in this assay, with ALK1-Fc as a positive control for the assay conditions. Both BMP9 and BMP10 were tested in this experiment. As shown in Fig. 7, being an effective ligand trap, ALK1-Fc showed a robust inhibitory effect on BRE-Luc induction in both cell lines and for both BMP9 and BMP10 treatments. Although sENG-Fc also binds both BMP9 and BMP10 with very high affinity, it only inhibited BMP9 and BMP10 activity in C2C12 cells, not in HMEC-1 cells, consistent with the results from the transfection experiments. This confirms that the effect of sENG on BMP9 (and BMP10) signaling is dependent on cell surface receptor context, and sENG can inhibit BMP9 (and BMP10) signaling in cells lacking ALK1 and ENG expression.

## Discussion

Previously, our groups have shown apparently opposite effects of sENG on BMP9 signaling. While sENG(D) inhibits BMP9 signaling in myeloma blood cancer cells, neither sENG(M) nor sENG(D) inhibits BMP9 signaling in wild type endothelial cells, albeit they did inhibit BMP9 signaling in *Eng*-/- endothelial cells. We hypothesized that such different effects of sENG on BMP9 signaling might be due to the cell surface receptor composition, particularly the presence or absence of ENG and ALK1. In this study, we first confirmed that different forms of sENG inhibited BMP9 signaling and activity in several non-endothelial cells, and then tested whether overexpression of ENG and ALK1 could modify the effect of sENG on BMP9 signaling. We found the most significant effect came from ALK1 transfection, where its overexpression suppressed the inhibitory effect of sENG on BMP9 activity. Finally we confirmed such findings using the BRE-Luc reporter assays in two cell lines, one endothelial and one non-endothelial, both stably transfected with the reporter gene BRE-Luc.

Different cell types express different receptors (type I, type II and co-receptors) and this determines which ligand can act on a given cell. Likewise, the affinities of ligands to receptors vary, and which ligand can bind and signal depends on multiple factors such as competition between ligands and the presence of antagonists and soluble receptors^5,26,28^. Like ENG, the BMP type I receptor ALK1 is predominantly expressed by endothelial cells^29^. BMP9 and BMP10 are the only described high-affinity ligands for ALK1 with dissociation constants in the picomolar range^30^, and were shown functionally equivalent in activating ALK1 in endothelial cells^31,32^. However, ALK2 has been shown to be a relevant type I receptor for BMP9 in various non-endothelial cells such as ovarian cancer cells, multiple myeloma cells, mesenchymal stem cells and myoblasts^8,19,20,33^. ALK2 was also suggested to have a role in mediating specific BMP9 actions in endothelial cells^34^. BMP10 can bind and signal via ALK3^35^. Both ALK1-Fc and all forms of sENG have high affinities for BMP9 and BMP10. Although ALK1-Fc can act as ligand trap and inhibit the signaling activity of BMP9 and BMP10, the effects of sENG are context-dependent, with cell surface ALK1 and ENG levels playing a critical role.

In agreement with structural data that ENG binding sites overlap with the type II but not the type I receptor binding sites^14^, we show here that sENG inhibited BMP9 binding to ActRIIA-Fc, but not to ALK1-Fc. Similar results were reported in the Biacore binding assay that sENG-Fc and BMP9 complex can bind to ALK1 but not to ActRIIB-Fc or BMPRII-Fc^15^.sENG was reported to result from the partial shedding of cell surface ENG^36,37^, and has been proposed as a marker to predict cardiovascular events in patients with chronic artery disease after percutaneous coronary intervention^38^. Increased plasma sENG levels were suggested as an indicator of cardiovascular alteration in hypertensive and diabetic patients^39^. Increased ENG expression was found in the left ventricle of human subjects with heart failure, and sENG treatment attenuates cardiac fibrosis in an in vivo model of heart failure (pressure-overload induced heart failure)^40^. Circulating sENG has also been shown as a sensitive marker of prognosis and function in group 1 PAH^41^.

The magnitude of changes in sENG concentrations in these cardiovascular diseases are generally small, usually from 4 to 6 ng/ml in healthy controls to 8–10 ng/ml in patients. However, in PE patients, the measured sENG levels are typically above 20 ng/ml and can be as high as 200 ng/ml^42^. Interestingly, in both PAH and PE it was found that cell surface ENG were also increased^41,42^. This raised the question whether the elevated sENG level is merely a biomarker for endothelial dysfunction and reflects the increased expression in the tissue, or such elevated sENG concentrations also have functional consequences. Of note, elevated sENG levels have been reported to play a causal role on the pathogenesis of PE^42^. In this study, we tested sENG concentrations ranging from around physiological range (4 ng/ml) to above and beyond the highest reported concentration range (1000 ng/ml), and we found most of the inhibitory effect of sENG requires concentrations much higher than the highest levels being measured in human patients. Collectively, our previous and current data suggest that increased levels of sENG in most cardiovascular diseases apart from PE are unlikely to have any major impact on BMP9 signaling. At high sENG concentrations such as in PE patients, especially those with HELLP (Hemolysis, Elevated Liver enzymes and Low Platelets) syndrome, elevated sENG might be able to inhibit BMP9 (and BMP10) signaling in cells with low ENG and low ALK1 expression, especially when acting together with another confounding factor such as the elevated levels of sFLT1. Such context-dependent activities of sENG and cell surface ENG support previous reports that pharmacological interventions of ENG function, either by increasing sENG concentration using sENG-Fc to beyond physiologically observed concentrations, or by administration of antibodies targeting ENG, such as TRC105, will impact on ENG-dependent BMP9 signaling and cellular functions. Thus our data here provide a framework for targeting the ALK1/ENG pathways for therapeutic purposes and support the importance of further research into the mechanism of ENG at the molecular and protein levels.

## Materials and methods

### Cell culture and reagents

The human multiple myeloma cell line (HMCL) INA-6 was a kind gift from Dr M. Gramatzki (University of Erlangen-Nurnberg, Erlangen, Germany)^43^, and the IH-1 HMCL was established in our laboratory^44^. The INA-6 cells were grown in RPMI-1640 (Sigma-Aldrich Norway, Oslo, Norway) supplemented with 2 mM L-glutamine (Sigma-Aldrich), hereafter termed RPMI, with 10% fetal calf serum (FCS). The IH-1 cells were grown in RPMI with 10% human serum (HS) (Department of Immunology and Transfusion Medicine, St. Olav’s University Hospital, Trondheim, Norway). Recombinant human interleukin (IL)-6 (Gibco, Thermo Fisher Scientific, Waltham, MA, USA) (1 ng/mL) was added to the growth medium of both myeloma cell lines. HMEC1-BRE cells were grown as described previously^45^. C2C12 murine myoblast cells were from ATCC and were grown in 10% FCS in DMEM supplemented with 2 mM L-glutamine. C2C12-BRE(BH) were kindly provided by Dr B.Herrera and Prof. G Inman^46^. HEK EBNA cells were cultured in DMEM supplemented with 10% fetal bovine serum (FBS). The cells were maintained in 37 °C in a humidified atmosphere containing 5% CO_2_ and tested regularly for mycoplasma. For experiments with HMCL, 2% HS in RPMI was used as medium, with IL-6 (1 ng/ml) added. The recombinant human proteins BMP9 (Cat.# 3209-BP), BMP10 (Cat.# 2926-BP), and sENG-Fc (Cat.# 6578-EN) and sENG(D) in Fig. 1A (Cat.# 1097-EN) were from R&D Systems (Bio-Techne, Abingdon, UK), whereas dimeric and monomeric sENG, sENG(D) and sENG(M), were generated as described previously^16^.

### BRE-luc reporter assays

Generation of stable INA-6 BRE-luc cells with the bi-directional luciferase reporter plasmid, pLentiX1-pBREFLrRL, was described earlier^24^. The plasmid reports on SMAD1/5/8 transcriptional activity as Firefly luciferase. C2C12 BRE-luc cells were generated the same way as INA-6 BRE-luc and single-cell cloned before use in reporter assays. The assays were performed as follows: 50,000 cells/well were seeded in 96 well opaque plates in 0.1% bovine serum albumin (BSA) in RPMI and treated as indicated in each figure. The plates were equilibrated to room temperature before addition of Britelite plus substrate (PerkinElmer Inc., Waltham, MA, USA) and luminescence was measured with Victor 1420 multilabel counter (PerkinElmer Inc.).

For neutralization studies, HMEC1-BRE or C2C12-BRE(BH) cells were seeded at 30,000 cells/well in 96-well plates and grown for 3 days followed by overnight starvation. HMEC1-BRE cells were starved in serum-free MDCB131 containing antibiotic/antimycotic, whereas C2C12-BRE(BH) cells were starved with DMEM containing 0.1% FBS and antibiotic/antimycotic. BMP9 or BMP10 (at 0.2 ng/ml for HMEC-1 cells and 10 ng/ml for C2C12 cells) were incubated with sENG-Fc (2.5 µg/ml) or ALK1-Fc (1 µg/ml) at 37 °C overnight in their respective serum-free media containing 0.5% (w/v) BSA before applying to the cells.

### Alkaline phosphatase (ALP) assay

Transfected C2C12 mouse myoblast cells were seeded at 25,000 cells/well in a 24-well plate and cultured until 80% confluent. Treatment mixtures containing BMP9 (10 ng/ml) with or without the addition of sENG monomer or dimer at indicated concentrations were prepared in DMEM supplemented with 0.25% FBS and applied to cells in the final volume of 1 ml for 65 h. Cells were then lysed in ice-cold 1% Triton X-100 in PBS and protein concentrations were measured using the DC™ Protein Assay Kit II (BioRad). ALP activity was measured by incubating 15–20 μg of cell lysate proteins with pNPP substrate (Sigma- Aldrich) in 0.1 M glycine buffer, pH 10.4, containing 1 mM MgCl_2_ and 1 mM ZnCl_2_ in dark at 37 °C for 30 min. The absorbance was read at 405 nm on a Microplate Reader.

### RNA extraction and quantitative reverse transcription-PCR (RT-qPCR).

HEK EBNA were seeded at 200,000 cells/well in a 6-well plate followed by transfection with 2 μg of plasmid and a further incubation for 24 h. Total RNA was extracted using RNeasy Mini Kit buffers (Qiagen, West Sussex, UK) and Silica Membrane Mini Spin Columns (EconoSpin) following the manufacturer’s instructions. Equal amounts of RNA (~ 1 μg) were then reverse transcribed into cDNA using a High Capacity Reverse Transcriptase kit (Applied Biosystems). qPCR was performed on QuantStudio 6 Flex Real-Time PCR System (Applied Biosystems) using SYBR Green JumpStart Taq ReadyMix (Sigma-Aldrich). Differences in gene expression are presented as the fold change relative to controls. The relative expression level of *ID1* (Forward: 5′-GACGGCCGAGGCGGCATG-3′, Reverse: 5′-GGGGA GACCCACAGAGCACG-3′) was calculated using the △△Ct method by normalizing to β2-Microglobulin (*B2M* Forward: 5′-CTCGCGCTACTCTCTCTTTCT-3′; Reverse: 5′-CATTCTCTGCTGGATGACGTG-3′) and presented as fold change relative to controls.

For INA-6 and C2C12 BRE-luc cells, RNA was isolated from cells using the RNeasy Mini Kit (Qiagen, Crawley, UK), and complementary DNA was synthesized from total RNA using the High Capacity RNA-to-cDNA kit. qPCR was performed using StepOne Real-Time PCR System and Taqman Gene Expression Assays (Applied Biosystems). The Taqman assays used were: *ENG* (Hs00923996_m1), *ID1* (Hs00357821_g1) and *GAPDH* (Hs99999905_m1).The comparative C_t_ method was used to estimate relative changes in gene expression with *GAPDH* as the housekeeping gene.

### Transfections

The myeloma cell line INA-6 was transfected using the Nucleofector device (Amaxa biosystems, Cologne, Germany) and Cell Line Nucleofector Kit R (Lonza, Basel, Switzerland), as previously described^47^. The pDisplay-ENG plasmid used to express full length HA-epitope-tagged endoglin was a kind gift from C. Bernabeu^48^. INA-6 cells transfected with pDisplay-ENG or empty control vector were maintained in selection media containing G418 (1 mg/mL) until the cells were used in experiments.

Transfection in C2C12 mouse myoblast cells and HEK EBNA cells was performed in 6-well plates using Lipofectamine LTX with Plus reagent (Thermo Fisher Scientific). Briefly, transfection medium in Opti-MEM (Thermo Fisher Scientific) was prepared with 2 μg of plasmid, together with 9 μl /reaction of Lipofectamine® LTX Reagent and 3 μl /reaction PLUS reagent before applied to cells. C2C12 cells were transfected for 24 h and then re-seeded for experiments. C2C12 BRE-luc cells were transfected with pcDNA3.1-control plasmid, pcDNA3-ALK1-HA (gift from P. ten Dijke), or pcDNA3-ALK2-HA (gift from A. Moustakas, Addgene plasmid #80,870; http://n2t.net/addgene:80870; RRID:Addgene_80870)^49^ using Lipofectamine™ 3000.

### ALK2 knockout cells

INA-6 ALK2 knockout (k.o.) cells and negative control cells were generated using CRISPR/Cas9 technology as recently described^50^.

### Cell viability assay

CellTiter-Glo (Promega, Madison, WI, USA) was used to measure ATP levels in metabolically active cells hence providing a readout of viable cells in culture. Cells were seeded in 96-well optical plates and treated as indicated. CellTiter-Glo reagent was added according to the manufacturer’s protocol and luminescence was determined using Victor 1420 multilabel counter (PerkinElmer Inc., Waltham, MA, USA).

### ELISA-based binding assay

The ELISA-based binding assay was performed as described previously^27^. In short, 96-well Nunc MaxiSorp plates were coated overnight at 4 °C with recombinant chimeric receptors ActRIIA-Fc or ALK1-Fc in PBS (1 μg/mL). Wells were blocked for one hour with 1% BSA in PBS at room temperature before addition of dilutions of BMP9, sENG-Fc, sENG(M), sENG(D) or combinations of these. Bound BMP9 was detected using reagents from BMP-9 DuoSet ELISA (R&D Systems). Optical density was determined using iMark Microplate Absorbance reader (Bio-Rad, Hercules, CA, USA).

### Western blotting

Cells were treated as indicated, washed with ice cold PBS and lysed for 20 min on ice. The lysis buffer contained 1% IGEPAL CA-630 (Sigma Aldrich, St Louis, MO, USA), 150 mM NaCl, 50 mM Tris–HCl (pH 7.5), protease inhibitor cocktail Complete Mini (Roche, Basel, Switzerland), 1 mM Na_3_VO_4_ and 50 mM NaF. Samples were fractionated on NuPAGE Bis–Tris gels with MOPS running buffer (Invitrogen, Carlsbad, CA, USA). Gels were blotted onto nitrocellulose membranes, blocked with 5% non-fat dry milk in Tris-buffered saline containing 0.01% Tween 20 (TBS-T) and incubated overnight with primary antibodies as indicated. Primary antibodies used were phospho-SMAD1/5 (Cell Signaling Technology, Medprobe Oslo, Norway, Cat# 9516, RRID: AB_491015, 1:1000 dilution) and GAPDH (Abcam, Cambridge, UK, Cat# Ab8245, RRID: AB_2107448, 1:30:000 dilution). Blots were washed with TBS-T before incubation for 1 h with horseradish peroxidase-conjugated secondary antibodies (DakoCytomation, Glostrup, Denmark). The blots were washed thoroughly with TBS-T before bands were detected using SuperSignal West Femto (ThermoFisher Scientific, Waltham, MA, USA) as luminescence substrate and LiCor Odyssey FC (LI-COR Biosciences, NE, USA).

For HEK EBNA cells, total cell proteins were harvested using SDS-lysis buffer (125 mM Tris (pH 7.4), 2% SDS, 10% glycerol) containing an EDTA-free protease inhibitor cocktail (Roche). Cell lysates were denatured and fractionated on 12% SDS-PAGE gels. Proteins were then transferred onto nitrocellulose membranes (GE Healthcare) by semi-dry blotting. Membranes were then blocked for an hour at room temperature (RT) with 5% (w/v) non-fat milk in TBS-T buffer before probing overnight at 4 °C with antibody against Endoglin (BD pharmingen, 555690, RRID:AB_396041, 1:1000 dilution) or ALK1 (R&D systems, AF370, RRID:AB_2222591, 1:1000 dilution). Following washing, blots were probed at RT for an hour with anti-mouse horseradish peroxidase antibody (P0447, Dako; 1:2000 dilution) or anti-goat horseradish peroxidase (HRP) secondary antibody (P0449, Dako; 1:2000 dilution). Blots were then incubated with enhanced chemi-luminescence reagent (GE Bioscience) and visualized by using X-ray film (GE healthcare).

### Statistics

Data analyses were performed using GraphPad Prism 9.0 (GraphPad Software). Results are shown as mean ± SEM. Statistical significance was analyzed using One-way or Two-way ANOVA as detailed in the figure legends. Values of *P* < 0.05 were considered statistically significant.

## Supplementary Information

Supplementary Information.

## Data Availability

All data generated or analyzed during this study are included in this published article (and its Supplementary Information files).
