# Supplementary material for: High concentrations of soluble endoglin can inhibit BMP9 signaling in non-endothelial cells
Source: Sci Rep. 2023 Apr 24;13:6639. doi: 10.1038/s41598-023-33352-3 (PMC10126157; doi:10.1038/s41598-023-33352-3)
Supplement: Supplementary file 1 — Supplementary Information. [file 41598_2023_33352_MOESM1_ESM.pdf]

High concentrations of soluble endoglin can inhibit BMP9 signaling in non-endothelial cells

Clara Andersson-Rusch[§,1,2], Bin Liu[§,3], Ingrid Quist-Løkken[1], Paul D Upton[3], Oddrun Elise Olsen[1], Hanne Hella[1], Xudong Yang[3], Zhen Tong[3], Nicholas W Morrell[3], Toril Holien[†,1,2,4,5] and Wei Li[†,3]

Supplementary Information

Supplementary Figure 1. Soluble endoglin (sENG) reduces BMP9- and BMP10-induced apoptosis in myeloma cell line IH-1. A. The myeloma cell line IH-1 was treated with BMP9 (0.5 ng/ml) or BMP10 (50 ng/ml) and increasing doses of either sENG(M) or sENG(D). Cell viability was measured after 48 hours using the CellTiter Glo ATP assay. The graphs represent Mean ± SEM from N = 3 independent experiments. One-way ANOVA analyses were performed in each BMP9 or BMP10 treated groups. **, P<0.01, ***, P<0.001, ****, P<0.0001. B. Uncropped images of western blots shown in Figure 1C.

Supplementary Figure 2. BMP9 is dependent on ALK2 to induce cell death in INA-6 cells. INA-6 ALK2 knockout cells and control cells were treated with BMP9 (0.125 ng/mL) with or without sENG-Fc (500 ng/ml). Cell viability was measured after 48 hours using the CellTiter Glo ATP assay. The experiments were performed four times, with duplicated measurement taken in each experiment. Data shown as Mean ± SEM. One-way ANOVA in each knockout cells, Tukey's multiple comparisons. **, P<0.01, ***, P<0.001.

Supplementary Figure 3. Optimization of ENG transfection conditions in HEK EBNA cells. Different amounts of pDisplay-ENG plasmid were tested in the transfection. Equal quantities of cell lysates were fractionated on a 12% SDS-PAGE and blotted with anti-ENG antibody (BD Pharmingen™, 555690). 1 μg plasmid was used in subsequent transfection experiments. Control: same amount of total cell lysate from PAECs.

Supplementary Figure 4. Optimization of ENG and ALK1 transfection conditions in C2C12 cells. Different amounts of pDisplay-ENG (**A**) and pcDNA3-ALK1-HA (**B**) plasmids were tested in the transfection. Equal quantities of cell lysates were fractionated on a 12% SDS-

PAGE under reducing conditions and blotted with anti-ENG antibody (**A**), or ALK1 antibody (R&D Systems, AF370) (**B**). For both ALK1 and ENG plasmid transfection, 2 μg of DNA was chosen for the subsequent transfection experiments. Control: same amount of total cell lysate from PAECs.

Supplementary Figure 1

A

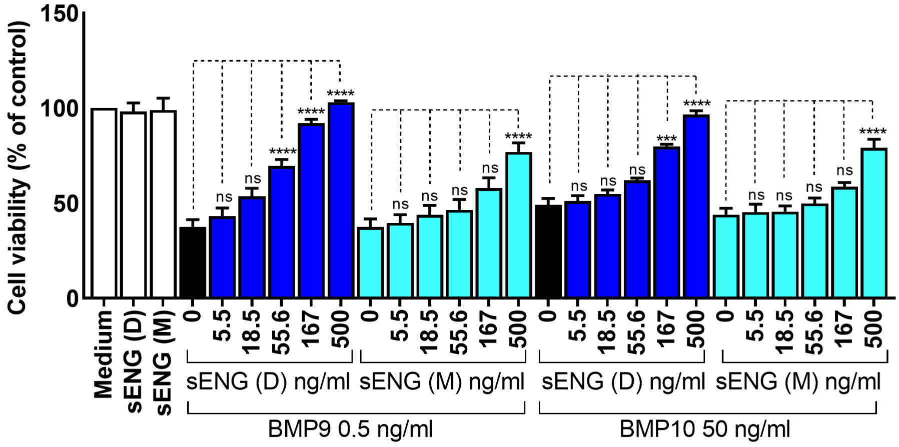

B

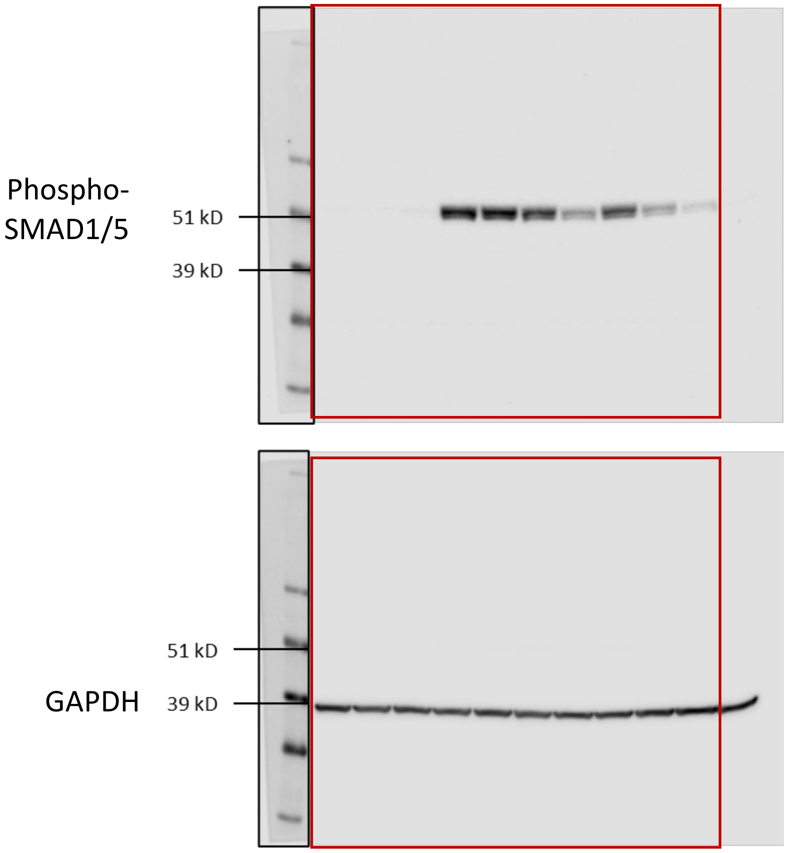

Supplementary Figure 2

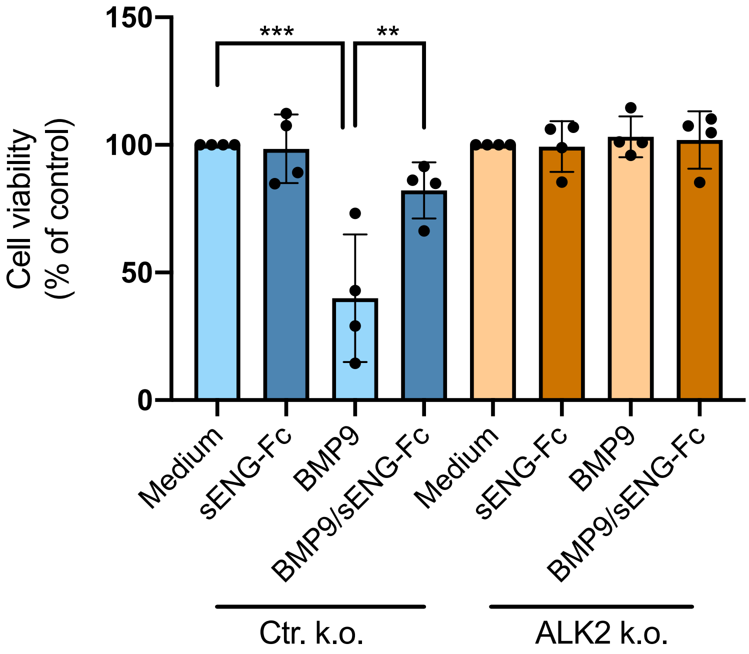

Supplementary Figure 3

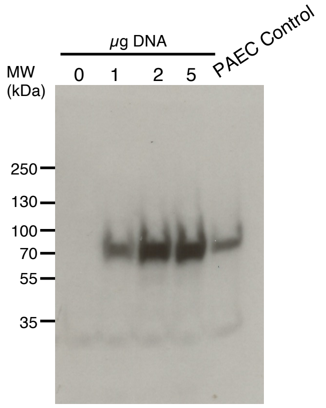

HEK cell, ENG transfection test

Supplementary Figure 4

A

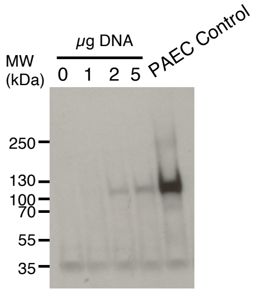

C2C12 cells
ENG transfection

B

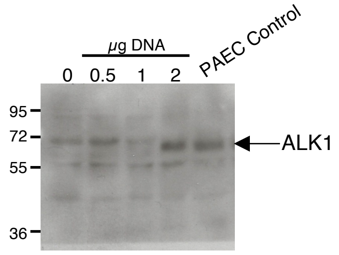

C2C12 cells
ALK1 transfection
